# Novel Ensemble Model Recommendation Approach for the Detection of Dyslexia

**DOI:** 10.3390/children9091337

**Published:** 2022-09-01

**Authors:** Ahmed Saeed AlGhamdi

**Affiliations:** Department of Computer Engineering, College of Computer and Information Technology, Taif University, P.O. Box 11099, Taif 21994, Saudi Arabia; asjannah@tu.edu.sa

**Keywords:** ensemble, dyslexia, machine learning, accuracy, disorder

## Abstract

There are a large number of neurological disorders being explored regarding possible management and treatment, with dyslexia being one of the disorders that affect children at the onset of their learning process. Dyslexia is a developmental neurological disorder that prevents children from learning. The disorder has a prevalence of around 10% across the globe, as reported by most of the literature on dyslexia. The early detection and management of dyslexia is one of the primary pursuits among different research. One such domain that leads this pursuit of the early detection and management of dyslexia is artificial intelligence. With so much effort being expended to explore the applicability of artificial intelligence to address the problem of dyslexia detection, in this work, an ensemble model for the early detection of dyslexia is proposed and recommend. The work experimentally considers a pool of ensembles with rigorous validation on a large sized dataset. The final ensemble model recommendation for detection is expressed after evaluating all of the ensemble frameworks based on a number of evaluation parameters. Our experiments reveal that the subspace discriminant ensemble showed superiority for the detection of dyslexia with an accuracy of 90% on five-fold cross validation with the least training time. An accuracy of 90.90% was achieved using boosted trees with a holdout validation of 30%, while with no validation the subspace K-Nearest Neighbor (KNN) outperformed the other ensembles with an accuracy of 99.9%.

## 1. Introduction

Dyslexia is a learning disability that occurs mostly in children during their early childhood, and it is one of the more common specific developmental disorders, with a prevalence of approximately 10% [1]. It will continue to grow if the early detection and management of dyslexia are not given full attention by people from different fronts who are both directly and indirectly involved in it. In Saudi Arabia itself, the percentage of incidence has already reached 7% and, as such, efforts are being made to detect the disorder early, so that management and preemptive measures are in place and on time [2]. Dyslexia is a disorder of a neurological origin that affects the learning of those who suffer from it. Dyslexic children face difficulties while reading, spelling, and writing words, despite having average or above-average intelligence; as a consequence, dyslexic children often suffer from negative feelings, such as low self-esteem, frustration, and anger. Therefore, the early detection of dyslexia is very important to support dyslexic children from as early as possible. The diagnosis of learning difficulties is a challenging goal. There are many factors that contribute to dyslexia. Family history is one such factor that results in dyslexia in children. Lower weight at time of birth or premature birth also contribute to dyslexia. Exposure to alcohol, drugs, or nicotine during pregnancy also contribute to the state of dyslexia in children.

The field of machine learning has evolved prospectively in the past few decades. Machine learning has solved a number of problems in a wide variety of domains, especially dyslexia [3]. These problems may include simpler problems or a complex set of problems: while simple problems can be addressed using machine learning in a very simplistic manner, complex problems demand the use of more sophisticated machine learning algorithms [4,5]. Machine learning algorithms tend to face numerous challenges, which mainly come from poor data quality, as well as over-fitting and under-fitting of the model, and thus there is always a demand for developing a machine learning algorithm that can handle complex data [6]. With conventional machine learning models using a single base classifier, the prediction accuracy is hampered. In such a scenario, this work pursued what is known as an ensemble model for machine learning. The goal of an ensemble model is to basically make predictions based on minimizing generalization errors [7]. Instead of using a single model, multiple models each working in a hierarchical manner are used. The soul objective being that there is no over fitting with the model and the weaknesses of weak learners are eliminated. Hence, a better generalization performance is expected in an ensemble computer compared with the generalization performance offered by a single machine learning model. An ensemble model is analogous to the use of multiple experts giving opinions about some issues [8]. The opinion received from a number of experts can be seen as a more robust opinion compared with an opinion received from a single expert. Although the detection of dyslexia through making use of machine learning classification models has been attempted, it is yet to be pursued using ensemble frameworks. The evidence supporting the use of ensemble methods for the detection of dyslexia comes from various related works. The studies in [9,10] used non-linear classifiers while training them with equal proportions of data for the detection of dyslexia. However, this resulted in the class imbalance problem, which negatively affects the performance in learning of algorithms and thus hinders the process of generalization for the trained models. This idea is further supported by the works of [11,12], which discuss the use of Artificial Neural Networks (ANN) for the classification of dyslexia. These works reveal that training done using this method is prone to overfitting due to its nature of making complex models that are not properly generalized. Based on these challenges, the authors explored the importance and applicability of ensemble models to detect the learning disability of dyslexia with a high performance rate.

The main contributions of this paper are as follows:

1. An efficient ensemble model is presented and developed for the early detection of learning disability in dyslexia.
2. An experimental model is developed for a pool of ensembles where validation is carried out rigorously.
3. The performance of the ensemble model recommendation for the detection of dyslexia is evaluated on the basis of metrics of accuracy, precision, recall, f-measure, and AUC.

This research article is structured as follows: Section 2 presents the related work. Section 3 provides the novelty of research. Section 4 provides the details related to the dataset used in this study. Section 5 provides the methodology for this work, which is inspired from the potential of machine learning’s ensemble modeling. The experimental findings and evaluation of the ensemble model recommended for early dyslexia detection are described in Section 6. Finally, the findings are concluded in Section 7.

## 2. Related Work

The proposed work attempts to explore the use of an ensemble model for the detection of dyslexia. The evolution of ensemble models started almost four decades ago. One of the earliest ensemble systems was proposed in [3] by Sheela and Dasarathy in 1979. The idea behind their work was partitioning of the feature set using a pool of classifiers. Work along the same line continued and a decade later, the authors of [13] pointed towards the utilization of an ensemble of closely configured NN for performance enhancement. The work implemented by [14] demonstrated that an ensemble can be created in which a random strong classifier with a fairly low error rate (for a binary classification) can be reconstructed using a sequence of weak classifiers whose error rates are fairly larger than the strong classifier. This aggregation was referred to as boosting by [15]. The evolution of the adaboost algorithm, in essence, came into existence with the concept of boosting. Adaboost is currently seen as one of the most popular ensemble methods for multiclass classification problems and regression [16]. From this point onwards, a significant amount of work on ensemble methods has been seen in the literature and has been given different names by many authors, such as composite classifier systems [6], mixture of experts (MoE) [17,18], stacked generalization [19], bagging [20], classifier fusion [21,22,23], consensus aggregation [24], dynamic classifier selection [25], committee of neural networks [26], classifier ensembles [27,28], combination of multiple classifiers [29,30,31,32,33], and many others. Most of the works on ensemble frameworks suggest that the selection of an ensemble is actually driven by one of the following factors: training data selection or training base classifiers and aggregating classifiers. The potential of ensemble modeling in predicting diverse diseases and disorders is still being exploited by different authors. In a recent work proposed by [34], the utilization of ensemble modeling in descriptive epidemiology is considered for the burden of disease estimation. The authors in [35] presented a cancer prediction methodology by utilizing deep learning with a multi-model ensemble method. Similarly, ensemble modeling was pursued by the authors in [36] for the early prediction of diabetic retinopathy. Recently, prediction for potential heart disease risk was carried out by the authors in [37].

On the other hand, significant work on dyslexia has been reported over time by different authors across the world. A descriptive study was made by [38] on the problems faced by dyslexic children in elementary underdeveloped countries. Different methods to adopt for the treatment of dyslexia have been suggested. The authors of [39] demonstrated eye-tracking technology for the analysis of dyslexia. Reading time and the number of fixations are two parameters that can be used to detect dyslexia. The authors of [40] made use of machine learning techniques that helped to identify dyslexia. By making use of machine learning techniques, classification of the disorder was achieved with a high degree of specificity. A mobile app was developed by [41], which helped children to distinguish between the letters b and d. This helps with alphabet recognition in a very exciting manner without the children being under pressure. A study was conducted by some of the professors from the University of Amsterdam [42] on women controls between 18–21 years. Structural changes were achieved in the grey matter by making use of neuro scans and it was also classified using SVM to obtain a 59% performance accuracy.

Most works on dyslexia detection are oriented towards the use of machine learning algorithms and some of the works are summarized in Table 1.

The development of classification models for the detection of dyslexia has received attention in past years with the evolution and use of machine learning, with promising accuracy. With diverse factors to be handled while choosing a machine learning framework for this task, previous studies have shown promise, with each of the machine learning models being driven mainly by ANN, KNN, SVM, and LR. To diagnose the condition of dyslexia in children, ensemble-based studies are uncommon in the literature. Based on the literature discussed in this section, the authors explored the importance and applicability of ensemble models to detect the learning disability of dyslexia with a high performance rate. Moreover, the authors attempted to recommend the best ensemble model for the detection of dyslexia by performing a series of cross-validation tests for the implemented model.

## 3. Novelty of Research

In this work, an ensemble model for the early detection of dyslexia is proposed and recommend. The work experimentally considers a pool of ensembles with rigorous validation on a large sized dataset. The final ensemble model recommendation for the detection is expressed after evaluating all of the ensemble frameworks based on a number of evaluation parameters. Our experiments reveal that the subspace discriminant ensemble shows superiority for the detection of dyslexia with an accuracy of 90% on five-fold cross validation with the least training time. An accuracy of 90.90% was achieved by using boosted trees with holdout validation of 30%, while with no validation, the subspace K-Nearest Neighbor (KNN) outperformed the other ensembles with an accuracy of 99.9%.

## 4. Dataset

**Figure 1:**
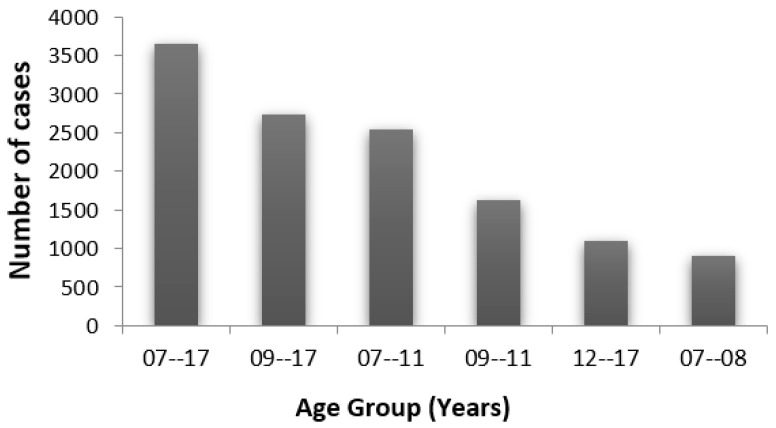
Case distribution for various age groups.

**Figure 2:**
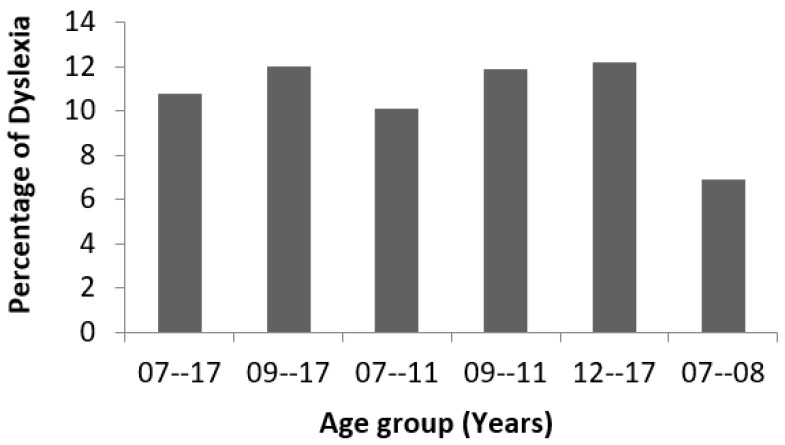
Age wise percentage of dyslexia.

The dataset chosen for this study uses a thorough evaluation of the following components of language speaking and understanding: phonological awareness, morphological awareness, visual discrimination and categorization, alphabetic awareness, syllabic awareness, semantic awareness, auditory discrimination and categorization, visual working-memory, and sequential auditory-working memory. The dataset is 3644 subjects with 196 attributes representing two classes: dyslexic and non-dyslexic. The dataset is freely available and can be accessed on https://doi.org/10.34740/kaggle/dsv/1617514 (accessed on 5 December 2021). Figure 1 below shows the distribution of cases with various age groups for this dataset. The subjects for this study consist of children whose ages fall in the range of 7 to 17 years. Similarly, Figure 2 shows the percentage of dyslexia for various age groups for this dataset. It is worth noting that the dataset has a nearly flat distribution.

## 5. Problem Formulation and Methodology

**Figure 3:**
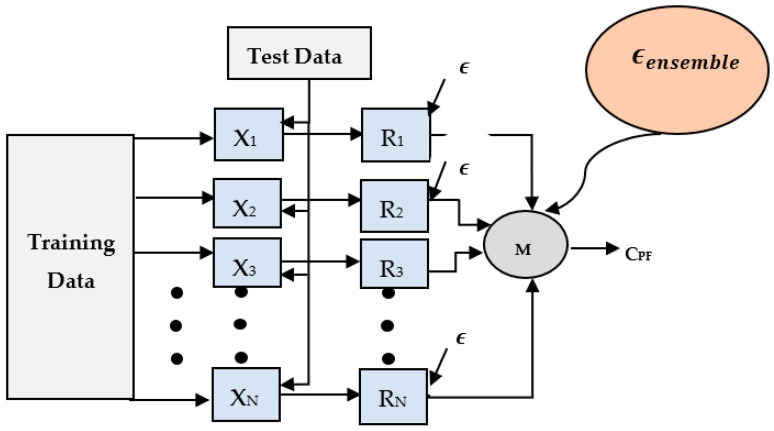
Error minimization using a number of base classifiers in an ensemble.

The methodology for this work is inspired from the potential of machine learning’s ensemble modeling. The rationale of choosing this work is illustrated in Figure 3. The main idea behind using ensemble modeling is error minimization in the framework of weak classifiers. For example, the simplest implementation of an ensemble can be accomplished via a majority voting from a pool of base classifiers.

With respect to Figure 3, let *X*_1_, *X*_2_, …, *X_N_* be a pool of classifiers, each making a prediction *R*_1_, *R*_2_, …, *R_N_*, respectively, for a given classification task. With each base classifier *X_j_* making prediction *R_j_*, a binary class label C_PF_ (Dyslexic (1) or Non-dyslexic (−1)) is chosen with maximum votes, as follows:

(1) $$\begin{array}{lll} b(i)=\mathrm{sign}\;\left[\overset{n}{\underset{j}{\sum }}X_{j}\left(i\right)\right] & \mathrm{Dyslexic}\;(1) & \mathrm{if}\underset{i}{\sum }B_{j}(i)\;\geq \;0\\ & \mathrm{Non-dyslexic}\;(-1) & \;\mathrm{Otherwise} \end{array}$$

Each base classifier *X_j_* imparts an error *E* during the classification process.

Assuming multiple classifiers, it can be seen that the cumulative error is reduced in an ensemble model. For example, with 9, 10, and 11 base classifiers for the current binary dyslexia classification, each performing a classification task with an error probability of 0.25, the ensemble error as given by Equation (2) is minimized to 0.165, 0.078, and 0.034, respectively, which is significantly less than the individual classifier’s error of 0.25.

(2) $$\begin{array}{l} P(C_{p}\geq k)=\overset{n}{\underset{k}{\sum }}\langle_{k}^{n}\rangle E^{k}{\left(1-0.25\right)}^{n-k}\\ P(C_{p}\geq k)=\;=\epsilon_{ensemble} \end{array}$$

**Figure 4:**
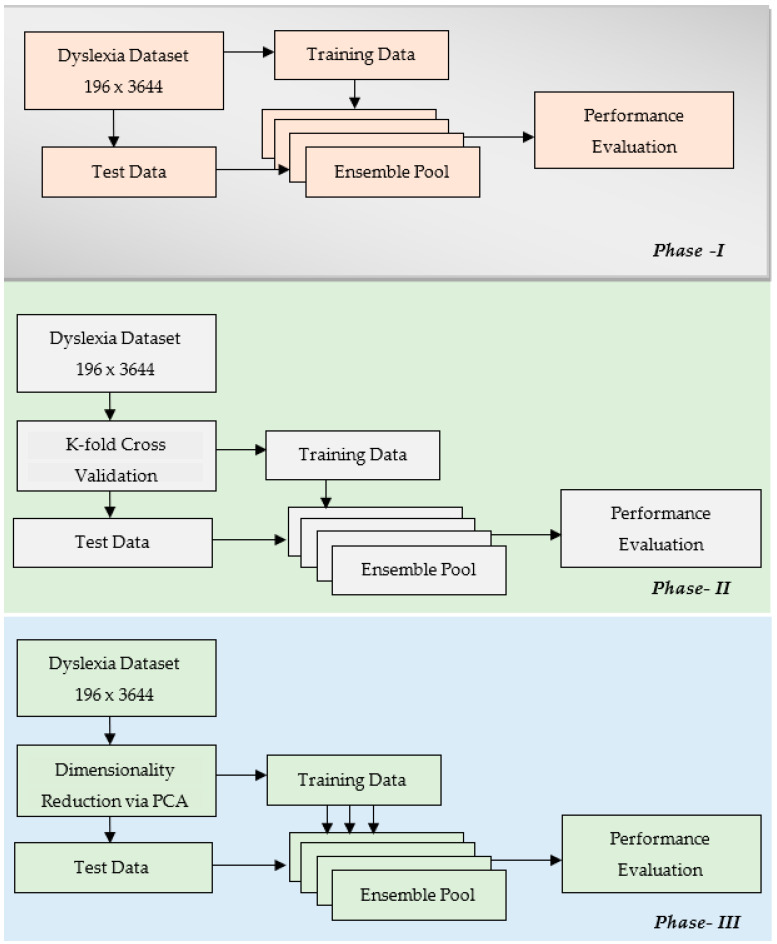
Schema for the proposed methodology.

Similarly, the more base classifiers, the lower the cumulative error of the whole framework of an ensemble. A low bias and low variance are highly desired while selecting a machine learning model for a classification task. As the bias and variance are related to each other in an inverse manner, there is a bias–variance tradeoff that needs to occur. In pursuit of making the best tradeoff, that is to say that minimizing both bias and variance, one common approach is to create an ensemble that is based on homogeneous base learners. As depicted from Equations (1) and (2), this framework reduces the overall error in an ensemble model. Considering this potential for an ensemble, this work aims to recommend an ensemble framework for the detection of dyslexia. Keeping in mind an alarming 15% incidence rate across the globe, it is imperative that an early detection framework be developed. Here, the proposed methodology was carried out in three phases, as shown in Figure 4.

The proposed schema consisted of mainly three phases. The first phase implemented an ensemble pool framework, wherein training and testing was done with all of the feature space available, 196 × 3644. The performance of this pool of ensembles was estimated using parameters of classification accuracy, ROC, and F1-score. In the second phase, all the feature space was fed into this pool of ensembles via a cross validation strategy aimed at minimizing the overfitting of the model. Different cross validations were performed using 5-fold, 10-fold, 15-fold, etc. The performance metrics were estimated in this phase for the 5-fold, 10-fold, and 15-fold cases. In the third phase, further modification was done in the way of feeding data into the pool of ensembles. A well performing dimensionality technique, viz. principal component analysis, was utilized to minimize the training time of the models. PCA was implemented in such a way that most of the data variance was retained. Having chosen a variance retention percentage of 95%, the pool of models was trained again and the performance was recorded.

As the major contribution of this research work, the best ensemble model based on the scheme of selection among ensemble models by performing a set of validation checks was proposed and recommended. The authors ensured that biasing of the recommended model was taken care of by avoiding any underfitted or overfitted situations. Bias–variance tradeoff was tuned for the recommended model by applying a series of cross validations as different stages of the model training. The following ensemble methods were incorporated in this work for the development and recommendation of pool of ensembles.

*1. Bootstrap Aggregating:* This technique allowed us to refine the model with a lower variance. In bootstrapping, data subsets were selected randomly from the training part of the dataset to take their predictions. Next, an approach of the weighted averaging was applied for the predictions of the datasets to boost the variance of the model. This technique played a vital role in ensemble learning by producing models that were diverse for random samples of the original dataset. This technique had a high tendency to help classifiers by avoiding overfitting, which resulted in a reduction of variance errors and bias. In general, bootstrap aggregating involves the following sequence of steps:

(*a*) Generation of a new training data set by random sampling with replacement from the original dataset.
(*b*) Training of the base learner with the training dataset, as achieved in step a.
(*c*) Base learners continuously trained with different subsets of datasets based on the pre-defined stopping criteria of steps a and b.
(*d*) Aggregation carried out by forming a final classifier from all of the base learner classifiers.

*2. Boosting:* This technique improves the predictions of the model by training the weaker classifiers sequentially and then correcting their predecessors until they are converted to strong classifiers. This technique is majorly focused on reducing bias. The improved accuracy using this ensemble was is achieved in a step-wise manner from the first to last base learner. In general, boosting involves the following sequence of steps:

(*a*) Equal weights assigned for each sample in the training data.
(*b*) The first base learner classifier is trained with the weighted training data.
(*c*) More weight is assigned for all the training instances that are misclassified by the first base learner. The updated training weight of the samples are used to train the next base learner.
(*d*) The training error is minimized with each proceeding step by updated weights of the training instances.
(*e*) Final classifier results by the aggregation of sequentially trained base learners with weights.

*3. Subspace:* In this technique, a separate feature space for each base learner was utilized, which trained each base learner using a subspace of the dataset and not the entire dataset. This ensured that the correlation among various features/subspaces was eliminated. Classifiers such as k-NN and discriminant classifier make use of the subspace ensemble learning method. In general, boosting involves the following sequence of steps:

(*a*) N subsets are chosen containing M number of random features out of D features.
(*b*) Use each random subset to train N weak learners.
(*c*) Apply majority voting for making the final prediction.

*4. RUSBoosting:* While dealing with skewed training data, the trained model may show high variance. The class imbalance problem can be resolved by using a technique called resampling. The resampling procedure may involve either oversampling or under sampling. The idea of under sampling is that the two classes in the training data are balanced by eliminating samples from the majority class. Similarly, oversampling implies adding synthetic samples of a minority class to balance out the training data. When the boosting framework utilizes under sampling at each base learner stage, it is referred to as RUSboosting. The implementation of RUSboost is similar to boosting with one additional procedure at each base learner training stage, which is to under sample the training data. This technique ensures a low error rate and high performance.

## 6. Experimental Results and Discussion

As ensemble frameworks have not been explored for the detection of dyslexia, we fundamentally aimed to determine the best possible ensemble that could be used for the early detection of dyslexia. The recommendation developed here was based on a step-by-step experimental formulation covering all of the tree aspects of ensemble recommendation. Initially, we have identified various ensemble settings such as Adaboost, Bagging, Subspace, and RUSBoost to be used in line with the learners such as Nearest Neighbors, Discriminant Learning, and Decision trees. We rigorously tested a pre-selected pool of ensembles through a validation framework on an Intel (R) Core (TM) i7-9750H CPU @ 2.60 GHz–2.59 GHz processor with 16 GB RAM, so that the best recommendation could be offered for dyslexia detection. The summary of preset values and methods is provided in Table 2.

The experimental setup was such that we tested pool of the ensemble’s models on various cross validation folds. The rationale for working with different cross validation folds was to determine the efficacy of these ensembles in view of the unseen data. The cross-fold validation was an essential parameter in building a machine learning model for a specific application. Another method used for estimating the generalization performance was the holdout. In this work, we chose a holdout percentage of 30%, which was approximately 1100 test cases.

As can be observed from Table 3, the subspace discriminant ensemble performed the best from the pool, with an accuracy of nearly 90% with five-fold cross validation. The detection accuracy in the case of boosted trees showed a gradual increase from 87.90% to 92.60% when going from 5-fold to 20-fold cross validation. The detection accuracy of the subspace discriminant ensemble remained fairly consistent during all of the validation experimentation, and thus depicted a stable model behavior. As expected, during no validation experimentation for the subspace KNN ensemble, detection accuracy was the highest with a value of 99.9%. This could be attributed to the fact that the model overfit the data and was unable to capture randomness, giving a 99.9% accuracy on training data. The same can be true for the rest of the ensembles with detection accuracies wavering around 78%, 88%, and 89% for RUS boosted, Subspace KNN, and bagged trees, respectively.

**Figure 5:**
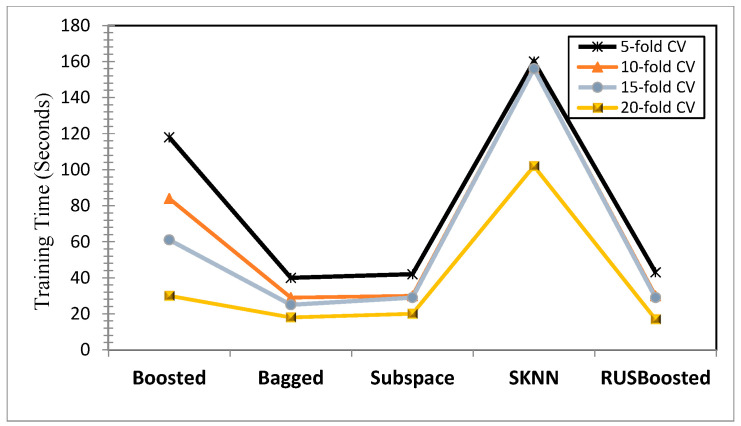
Training time comparison for various ensemble models with different CVs.

**Figure 6:**
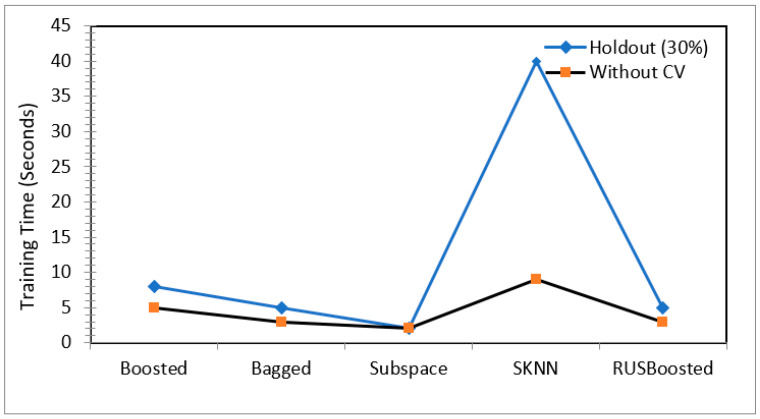
Training time comparison for various ensemble models for the holdout method and with no cross-validation.

Figure 5 and Figure 6 show the training time for all of the ensembles as per the setting offered in Table 3. Experiments reinforced the point that for most of the ensemble models, the training time increased when the “k” in the k-fold cross validation increased. As can be observed in Figure 5, boosted trees showed a significant increase in training time from 28 s (five-fold cross validation) to 81 s (20-fold cross validation). For the bagged tree, subspace discriminate, and RUS boost tree ensemble, the training time remained in the range of 20 s to 40 s for all of the cross-validation tests, which thus depicted their minimal dependence on the training/validation pattern. Out of all of the ensemble models, subspace KNN worked with the highest training time in the range of 100 s to 160 s. Figure 6 shows the training time of a pool of these ensembles with holdout validation (30%) and with no validation.

**Figure 7:**
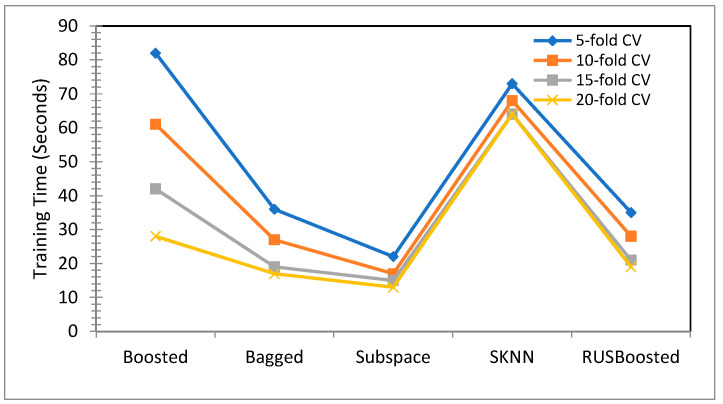
Training time comparison on various cross validations after performing the dimensionality reduction.

**Figure 8:**
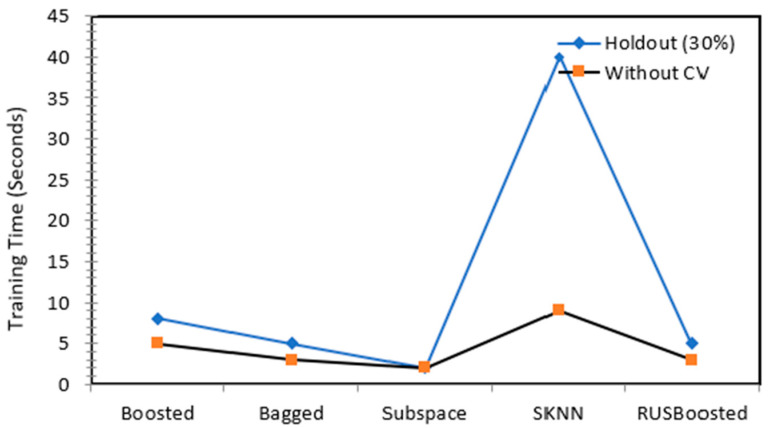
Training time comparison between the holdout method and no validation after performing the dimensionality reduction.

The next phase of the work was to carried out to test all of these ensembles with some pre-processing of the original dataset in order to reduce the training time. The pre-processing was carried in the form of dimensionality reduction of the data. As the original dataset was 196 × 3644 in size, this work reduced it to 43 × 3644 by PCA. The number of components was set to 43, keeping in view the variance that was being retained. For this work, variance of 95% from the original data was retained and this retention came from retaining the first 43 principal components. The results of this setting can be observed from the Table 4, wherein all of the detection accuracies for the ensembles with RUS boosted trees performing poorly with an average accuracy of 67% are recorded. The subspace discriminant ensemble remained stagnant with a detection accuracy of 89.20%. Moreover, the training time was reduced for most of the ensembles, as shown in Figure 7 and Figure 8, with the subspace showing a training time in the range of 12 s to 25 s, which was a significant improvement.

The different validation parameters used in the binary classification are tabulated in Table 5. The parameters included precision, recall, F1 score, area under curve of ROC, and accuracy. Moreover, the impact on the number of learners on these parameters is tabulated in Table 6. The trend indicates the improvement of validation parameters with the increase in the number of learners.

The subspace discriminant ensemble showed superiority for the detection of dyslexia with an accuracy of 90% on five-fold cross validation with the least training time. An accuracy of 90.90% was achieved using boosted trees with holdout validation of 30%, while with no validation subspace, the K-Nearest Neighbor (KNN) outperformed the other ensembles with an accuracy of 99.9%.

## 7. Conclusions

In an attempt to pursue the early detection of dyslexia, the proposed work aimed to explore the utility of ensemble models in the detection of dyslexia. The detection accuracy of the subspace discriminant ensemble remained fairly consistent during all of the validation experimentations, which depicted a stable model behavior. As could be expected, when there was no validation experimentation for the subspace discriminant ensemble, the detection accuracy was the highest with a value of 90.10%. The experiments depict that subspace discriminate ensemble showed superiority in terms of dyslexia detection and a minimal amount of training time. The ensemble showed a consolidated predicted outcome after rigorous validation tests on a large dataset. Moreover, the work shows that dimensionality reduction plays a role in formulating an optimal prediction model. The number of learners and the learning rate helped us tune the model for the best accuracy and optimality.

## Figures and Tables

**Table 1:** Summary of related works on dyslexia detection.

Reference	Age Group	Baseline Language	Dataset Size	ML Technique Used	Modality Used
[40]	18 and above	English	32	SVM	EEG
[41]	6–7	Hebrew	32	SVM NN	EEG
[42]	8	Dutch	44	SVM KNN	EEG
[43]	11–55	Spanish	97	SVM	Eye Tracking
[44]	9–10	Swedish	185	SVM	Eye Tracking
[45]	8–13	Greek	69	SVM NB K Means	Eye Tracking
[46]	10–15	Mandarin	61	SVM LR	MRI
[47]	8–14	German Polish French	236	SVM RF LR	MRI

**Table 2:** Preset values and methods in the experiments for an ensemble selection.

Operating Ensemble	Ensemble Method	Learning Method
Ensemble 1	Adaboost	Decision Trees
Ensemble 2	Bagging	Decision Trees
Ensemble 3	Subspace	Discriminant
Ensemble 4	Subspace	Nearest Neighbors
Ensemble 5	RUSBoost	Decision Trees

**Table 3:** Accuracy of ensemble models without dimensionality reduction.

Model	Ensemble Models					Validation
	Boosted Trees	Bagged Trees	Subspace Discriminant	Subspace KNN	RUS Boosted Trees	
Accuracy	87.90%	89.60%	89.80%	88.80%	78.00%	5-fold cross validation
Accuracy	90.50%	89.80%	89.70%	88.60%	77.90%	10-fold cross validation
Accuracy	90.30%	89.70%	89.70%	88.70%	78.90%	15-fold cross validation
Accuracy	90.20%	89.80%	89.80%	88.90%	78.40%	20-fold cross validation
Accuracy	90.90%	89.70%	89.40%	88.30%	77.90%	Holdout (30%)
Accuracy	92.60%	99.70%	90.10%	99.9%	82.40%	Without CV
Average Accuracy	90.40%	91.38%	89.75%	90.55%	78.92%	-

**Table 4:** Ensemble accuracies with dimensionality reduction versus CV.

Model	Ensemble Models					Validation
	Boosted Trees	Bagged Trees	Subspace Discriminant	Subspace KNN	RUS Boosted Trees	
Accuracy	89.10%	89.10%	89.20%	84.60%	66.70%	5-fold cross validation
Accuracy	89.20%	88.90%	89.20%	83.90%	68.10%	10-fold cross validation
Accuracy	89.30%	89.30%	89.20%	84.30%	67.10%	15-fold cross validation
Accuracy	89.10%	88.90%	89.20%	84.40%	68.60%	20-fold cross validation
Accuracy	88.80%	89.00%	89.20%	83.90%	61.50%	Holdout (30%)
Accuracy	90.10%	99.70%	89.20%	99.9%	72.60%	Without CV
Average Accuracy	89.27%	90.82%	89.20%	86.85%	67.43%	-

**Table 5:** Various validation parameters on five-fold CV.

Ensemble Method	Precision	Recall	F1 Score	AUC	Accuracy
Adaboost	0.98	0.91	0.94	0.84	90.3
Bagging	0.99	0.90	0.94	0.80	89.8
Subspace Discriminant	0.98	0.90	0.94	0.82	89.7
Subspace kNN	0.97	0.90	0.93	0.74	88.9
RUSBoost	0.90	0.97	0.93	0.74	78.7

**Table 6:** Effect of the number of learners on various validation parameters.

No. of Learners.	Precision	Recall	F1 Score	AUC	Accuracy
5	0.98	0.90	0.94	0.73	89.6
10	0.98	0.90	0.94	0.79	90.3
15	0.98	0.90	0.94	0.82	90.1
20	0.98	0.90	0.94	0.83	90.3
25	0.98	0.91	0.94	0.84	90.4
30	0.98	0.91	0.94	0.84	90.3

## Data Availability

Data will be made available based on reasonable request to the corresponding author.
